# UCHL1 and Proteasome in Blood Serum in Relation to Dietary Habits, Concentration of Selected Antioxidant Minerals and Total Antioxidant Status among Patients with Alzheimer’s Disease

**DOI:** 10.3390/jcm11020412

**Published:** 2022-01-14

**Authors:** Sylwia Bogdan, Anna Puścion-Jakubik, Katarzyna Klimiuk, Katarzyna Socha, Jan Kochanowicz, Ewa Gorodkiewicz

**Affiliations:** 1 Bioanalysis Laboratory, Faculty of Chemistry, University of Bialystok, Ciołkowskiego 1K, 15-245 Bialystok, Poland; sylwiabogdan93@gmail.com (S.B.); ewka@uwb.edu.pl (E.G.); 2 Department of Bromatology, Faculty of Pharmacy with the Division of Laboratory Medicine, Medical University of Białystok, Mickiewicza 2D Street, 15-222 Bialystok, Poland; katarzyna.socha@umb.edu.pl; 3 Podlasie Center of Psychogeriatrics, Swobodna 38 Street, 15-756 Bialystok, Poland; litwk@wp.pl; 4 Department of Neurology, Medical University of Białystok, M. Skłodowskiej-Curie 24a Street, 15-276 Bialystok, Poland; kochanowicz@vp.pl

**Keywords:** Alzheimer’s disease, UCHL1, proteasome, nutrition, antioxidants elements, drug therapy

## Abstract

Alzheimer’s disease (AD) is an incurable neurodegenerative disease. It is the most common form of dementia among the elderly population. So far, no effective methods of its treatment have been found. Research to better understand the mechanism of pathology may provide new methods for early diagnosis. This, in turn, could enable early intervention that could slow or halt disease progression and improve patients’ quality of life. Therefore, minimally invasive markers, including serum-based markers, are being sought to improve the diagnosis of AD. One of the important markers may be the concentration of UCHL1 and the proteasome in the blood serum. Their concentration can be affected by many factors, including eating habits. This study was conducted in 110 patients with early or moderate AD, with a mean age of 78.0 ± 8.1 years. The patients were under the care of the Podlasie Center of Psychogeriatrics and the Department of Neurology (Medical University of Białystok, Poland). The control group consisted of 60 healthy volunteers, matched for gender and age. The concentration of UCHL1 and the 20S proteasome subunit were measured by surface plasmon resonance imaging (SPRI). In addition, a nutritional interview was conducted with patients with AD, which assessed the frequency of consumption of 36 groups of products. In the group of patients with AD, compared to the control group, we showed a significantly higher concentration of UCHL1 (56.05 vs. 7.98 ng/mL) and the proteasome (13.02 vs. 5.72 µg/mL). Moreover, we found a low negative correlation between UCHL1 and the proteasome in the control group, and positive in the AD group. The analysis of eating habits showed that the consumption of selected groups of products may affect the concentration of the tested components, and therefore may have a protective effect on AD.

## 1. Introduction

Alzheimer’s disease (AD) is a progressive, incurable neurodegenerative disease that is the most common form of dementia in the elderly. The causes of this disease are still not fully understood, and there are no effective methods of its treatment. The main pathogenesis of AD is the amyloid theory, according to which the main phenomenon is the deposition of Aβ42 peptide (42 amino acid amyloid protein) in the brain, which causes the death of neurons and the development of clinical symptoms of dementia. Secondary to amyloidosis are increased levels of oxidative stress, chronic inflammatory response, disturbances in mineral components metabolism homeostasis, formation of pathological ion channels, or changes in the transmission of neurotransmitters [1]. The second disease the process behind the development of AD, in addition to beta-amyloid (β-amyloid) deposition, is the formation of the so-called neurofibrillary ganglia, which contain double-helical filaments composed of the abnormally phosphorylated tau protein. In the process of abnormal transformations involving β- and γ-secretases, it is fragmented into insoluble forms of β-amyloid, which is deposited inside and then extracellularly in the form of senile plaques. β-amyloid is also deposited in the walls of small cerebral vessels in the cortical layer, and this process may lead to the formation of microbleeding foci located in the cortical structures. The number and distribution of senile plaques and neurons with signs of neurofibrillary degeneration are the basis for the neuropathological classification of AD diagnosis. The above pathological processes result in a decrease in the level of messenger substances, of which the reduction of acetylcholine content is the most important for the memory system [2].

Among the pathomechanisms involved in the process of neuronal death, there are also disorders in mitochondrial function, an abnormal reaction to oxidative stress, activation of cytokines and other pro-inflammatory factors, disorders in the systems of neurotrophins and their receptors [3,4].

So far, no effective treatment for AD has been developed. Only symptomatic treatment is used, which includes drugs affecting cognitive functions, including acetylcholinesterase inhibitors (donepezil and rivastigmine registered in Poland) and memantine, which is an antagonist of the NMDA receptor (N-methyl-D-aspartic acid), as well as antidepressant, antipsychotics, and anti-anxiety drugs. The causal treatment of AD is still in clinical trials. It should be used in the period when the loss of neurons in strategic areas for the disease does not exceed 50%. That is why it is so important to identify patients in the initial period of the disease in order to inhibit the neurodegenerative process and the full symptoms of the disease. Targeted treatments are available to prevent the toxic effects of two key proteins in AD: β-amyloid and tau. These are either anti-toxic Aβ antibodies or β-secretase and γ-secretase inhibitors, which are pathological enzymes involved in the amyloid cascade [5].

Enzymes such as E1, E2, and E3, deubiquitinating enzymes (DUBs) involved in UPS regulate disease-causing proteins by controlling the degree of ubiquitination. An interesting issue is the development of therapies that target enzymes in degenerative diseases such as AD [6,7,8]. Studies have shown that the ubiquitin-proteasome system (UPS), the major intracellular system for protein quality control in eukaryotic cells, is associated with the pathogenesis of AD. Growing evidence points to a close relationship between β-amyloid and UPS. Impairment of the UPS system in AD can degrade β-amyloid and lead to abnormal accumulation. At the same time, β-amyloid inhibits proteasome activity and disrupts the multi-vesicular body (MVB) sorting pathway, creating an interaction between β-amyloid and UPS. Mutant ubiquitin (Ub) and ubiquitin-1 like ubiquitin (UBL) are associated with β-amyloid accumulation. Meanwhile, E2 coupling enzymes, E3 ligases, and deubiquitinating enzymes play a key role in the proteasomal degradation of β-amyloid. The ubiquitin-proteasome system has a profound effect on the amyloidogenic pathway of processing the amyloid precursor protein (APP) that generates β-amyloid. Upregulation of proteasomal degradation of BACE1 and gamma-secretase components leads to reduced accumulation of β-amyloid. An in-depth look at the mechanism underlying the interaction between β-amyloid and UPS could provide alternative therapeutic targets and lead to the development of new drugs and therapies [9].

Ubiquitin carboxy-terminal hydrolase L1 (UCHL1) is a deubiquitinating enzyme (223 amino acid protein encoded by 9 exons) [10]. This enzyme is found in all nerve cells in the brain (accounts for 1–2% of total brain protein) as well as ovary and testis [11,12]. AD and Parkinson’s disease are related to dysregulation of UCHL1 [13,14]. Loss of the amount of UCHL1 is occurred in amyotrophic lateral sclerosis and AD patients [14,15]. A decrease in the amount of UCHL1 increases the tendency of pancreatic beta-cells to programmed cell death. This protein protective function in neuroendocrine cells and explain the connection between diabetes and neurodegenerative diseases [16]. UCHL1 products hydrolyze small C-terminal adducts of ubiquitin to produce the ubiquitin monomer. This enzyme has both hydrolase and ligase activities [17]. Hydrolase activity deletes and converts ubiquitin molecules from degraded proteins. Moreover, it is also associated with proteasomal activity [18]. Ligase activity combines ubiquitin molecules for use in tagging proteins to delete [12]. Inactivation of the deubiquitinating enzyme inhibits ubiquitin-mediated proteolysis when the free ubiquitin will be spent or and saturation of the proteasome with polyubiquitin chains. Moreover, deubiquitination also plays a specific regulatory role. UCHL1 is relevant for maintaining free ubiquitin pool and for the proper function of the ubiquitin-proteasome system since inhibition of UCHL1 causes a 50% reduction of free ubiquitin in vitro [17]. In AD, the process of ubiquitination is crucial due to the fact that neurofibrillary tangles are positive for immunostaining to ubiquitin [19,20].

The proteasome is a protein complex that plays the essential role of deleting damaged or unnecessary proteins by proteolysis (chemical reaction is the breakdown of proteins into smaller polypeptides or amino acids). It is believed that the proteasome is the main component in the protein degradation pathway [21]. Proteasomes are found in the plasma of patients suffering from autoimmune and inflammatory diseases [22,23,24]. Interestingly in inflammatory conditions, the concentration of proteasomes in the blood correlates with the activity of the disease [25]. It has been observed that proteasome inhibition is a mediator of increased concentrations of aggregated protein, oxidized protein, and neuronal death in the brains of AD patients [26]. Studies show that proteasome activity is decreased in the brains of these patients [19,27,28,29,30].

The objective of the study was to estimate the concentration of UCHL1 and proteasome in patients with AD in terms of their clinical condition, dietary habits, smoking cigarettes, and selected indicators of oxidative stress.

## 2. Materials and Methods

### 2.1. Patients Characteristic

The study was conducted among 110 patients aged 54 to 93, under the care of the Podlasie Psychogeriatry Center in Białystok and the Department of Neurology, Medical University of Białystok, Poland. Patients had early or moderate AD diagnosed by geriatrician, according to the criteria of the National Institute on Aging-Alzheimer’s Association workgroups [31].

The control group consisted of 60 healthy people aged 52 to 83, without any cognitive impairment, most of whom were still working. The exclusion criteria were as follows: comorbidities such as type 1 and type 2 diabetes, autoimmune diseases, and cancer. The clock drawing test and mini-mental state exam (MMSE) scale were used to assess the severity of the disease. It included tests of memory, language, orientation, attention, and visual-spatial skills on a scale of 0 to 30 points. Table 1 presents the characteristics of the studied group.

The protocol of the study was approved by the Local Ethical Committee (R-I-002/210/2018).

All participants gave their written consent to participate in this study.

### 2.2. Eating Habits

Food frequency questionnaires (FFQ) developed by the Committee of Human Nutrition Sciences of the Polish Academy of Sciences were used to collect data. In order to assess their eating habits, patients with AD or their caregivers completed a questionnaire concerning the frequency of consumption of 36 food groups. The product groups and the criteria for assessing the frequency of consumption were described in detail in the previous publication [32].

### 2.3. Collection of Blood

Blood (approximately 6 mL) was collected from each participant in the study using a vacutainer tube containing a clot activator (Becton Dickinson, Rungis, France). The samples were then centrifuged (10 min, at approximately 1000× *g*). Serum samples were taken and stored frozen (at −20 °C).

### 2.4. Determination of UCHL1 and 20S Proteasome Concentration

The UCHL1 and proteasome concentrations were assessed using Surface Plasmon Resonance Imaging (SPRI).

The ubiquitin carboxy-terminal hydrolase L1 (human recombinant UCH-L1, R&D System. Inc., Minneapolis, MN, USA) concentration was measured by the SPRI biosensor. All of the necessary steps in the preparation and optimization of the biosensor have been described in publication Matuszczak et al. and Sankiewicz et al. [17,33]. Gold chips were manufactured as described in other papers [34,35]. The gold surface of the chip was covered with photopolymer and hydrophobic paint. Chips were rinsed with ethanol and water and dried under a stream of nitrogen. They were then immersed in 20 mM of cysteamine ethanolic solutions for at least 2 h and after rinsing with ethanol and water dried again under a stream of nitrogen. The rabbit monoclonal IgG2A antibody specific for human UCHL (R and D System. Inc.) was immobilized on the thiol monolayer under suitable conditions. The antibody solution in a PBS buffer was activated with NHS (250 mM) and EDC (250 mM). Activation of the antibody was carried out by adding the mixture of NHS and EDC (1:1) in a carbonate buffer solution (pH 8.5) into the antibody solution and with vigorous stirring for 5 min at room temperature. 3 μL of this solution was placed on the active places with the amine-modified surface, and incubated at 37 °C for 1 h. After this time the biosensor was rinsed with water. Next, serum samples (10× diluted) were placed directly on the prepared biosensor. The volume of the sample applied on each measuring field was 3 μL. The time of the interaction with the antibody was a max of 10 min. The biosensor was washed with water and HBS-ES buffer solution pH = 7.4 (0.01 M 4-(2-hydroxyethyl) piperazine-1-ethane sulfonic acid, 0.15 M sodium chloride, 0.005% Tween 20, 3 mM EDTA), BIOMED, Lublin, Poland) to remove unbound molecules from the surface. UCHL1 concentration in the samples (after appropriate dilution) was read from the calibration curve prepared in the range of 0.1–2.0 ng/mL.

The 20S proteasome concentration was determined using the biosensor for the 20S proteasome. PSI inhibitor was used as a receptor [36].

PSI inhibitor (Z-Ile-Glu(OBut)-Ala-Leu-H)) at a concentration of 80 nM was activated with NHS (50 mM) and EDC (200 mM) in a carbonate buffer (pH = 8.5) environment and then placed on the thiol (cysteamine)-modified surface and incubated at 37 °C for 1 h [33]. After receptor immobilization, the biosensor was rinsed with water. Next, blood plasma samples (10× diluted) were placed directly on the prepared biosensor. The volume of the sample applied on each measuring field was 3 μL. The time of the interaction with the receptor was max maxing 10 min. The biosensor was washed with water and HBS-ES buffer solution pH = 7.4 (0.01 M 4-(2-hydroxyethyl) piperazine-1-ethane sulfonic acid, 0.15 M sodium chloride, 0.005% Tween 20, 3 mM EDTA), BIOMED, Lublin, Poland) to remove unbound molecules from the surface. 20S proteasome concentration was evaluated from the calibration curve of 20S proteasome prepared in the range 1.4–7.0 µg/mL.

SPRI measurements for the determination of UCHL1 and 20S proteasome concentration were performed as described in the previous papers and schematic diagram apparatus is given in the paper [37]. As controls of the level of nonspecific binding, some of the places on the biosensor covered with buffer were used. The SPRI signal was measured at a fixed SPR angle on the basis of registered images. The first image after immobilization of the antibody or inhibitor was taken. Then, the second image after interaction with UCH-L1 or 20S proteasome was taken. The SPRI signal was obtained by subtraction of the signal after and before interaction with a biomolecule, for each spot separately. The contrast values obtained for all pixels across a particular sample single spot were integrated. Then, the SPRI signal was integrated over the spot area. NIH Image J version 1.32 software was used to evaluate the SPRI images in 2D form and to convert of numerical signal to a quantitative signal.

### 2.5. The Relationship between UCHL1 and Proteasome Concentration vs. Total Antioxidant Status and Antioxidant Minerals Concentration

Determination of TAS and the content of antioxidant elements was carried out in accordance with the methodology described in detail in the previous publication. We assessed the relationship between the concentration of UCHL1 and proteasome vs. the concentration of selected antioxidant elements and TAS, which were published earlier [32].

### 2.6. Statistical Analysis

Statistical analyzes were performed using Statistica v.13.3 (TIBCO Software, Inc., Palo Alto, CA, USA). The normality of the data distribution was assessed using the Shapiro-Wilk and Kolmogorov-Smirnov tests. U Mann-Whitney or ANOVA Kruskal-Wallis tests were used to assess the differences between the groups. In addition, the strength of the correlation was assessed using Spearman’s rank test. Stepwise multiple linear regression was used to determine the impact of eating habits on the content of elements and TAS in the studied patients. Differences at the level of significance *p* < 0.05 were considered statistically significant.

## 3. Results

### 3.1. Concentration of UCHL1

A significantly higher median concentration of UCHL1 was found in the study group as compared to the control group (56.05 vs. 7.98 ng/mL, *p* < 0.000001) (Table 2).

Moreover, there was no correlation between the concentration of UCHL1 and the result of the MMSE test, the result of the clock drawing test, age, and BMI.

**Figure 1:**
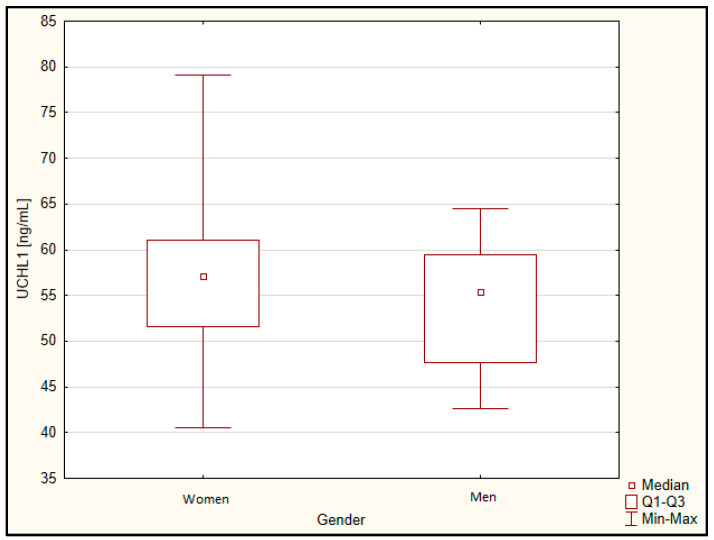
Concentration of UCHL1 depending on gender. Q1, lower quartile; Q3, upper quartile.

**Figure 2:**
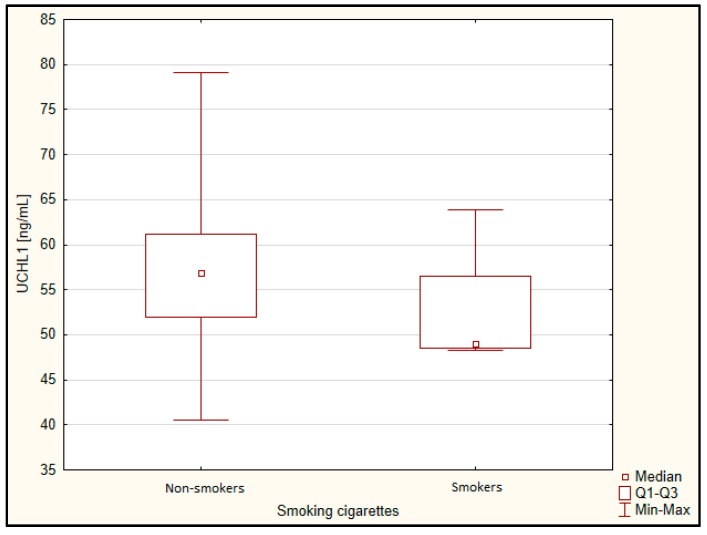
Concentration of UCHL 1 depending on smoking cigarettes. Q1, lower quartile; Q3, upper quartile.

There was also no difference by gender (Figure 1) and smoking status (Figure 2).

Based on the MMSE result, patients were divided into two categories (mild and moderate dementia) and five categories (profound dementia, moderate dementia, mild dementia, cognitive impairment without dementia, normal). There was no difference in the concentration of UCHL1 between the groups.

There was no significant correlation between the concentration of UCHL1 and the concentration of Se, while significant correlations are presented in Table 3.

We showed that the type of pharmacotherapy used did not affect the level of UCHL1 (Table 4).

### 3.2. Concentration of Proteasome

Patients with AD had a higher proteasome concentration compared to the control group (13.02 vs. 5.72, *p* < 0.0001) (Table 5).

**Figure 3:**
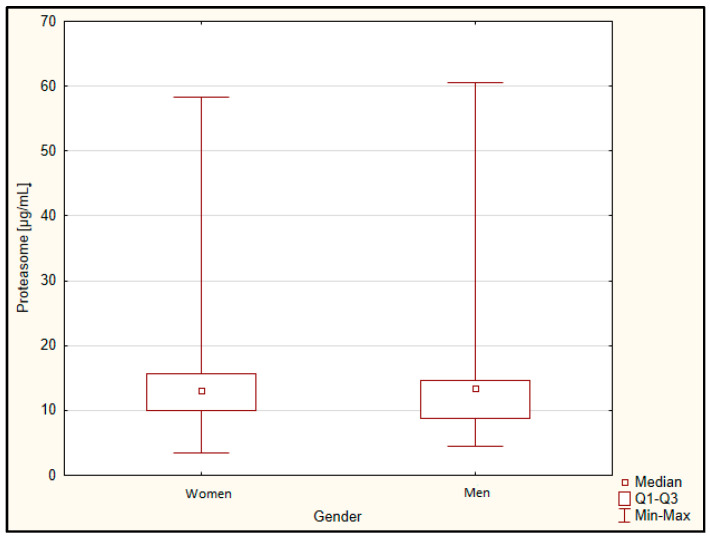
Concentration of proteasome depending on gender. Q1, lower quartile; Q3, upper quartile.

**Figure 4:**
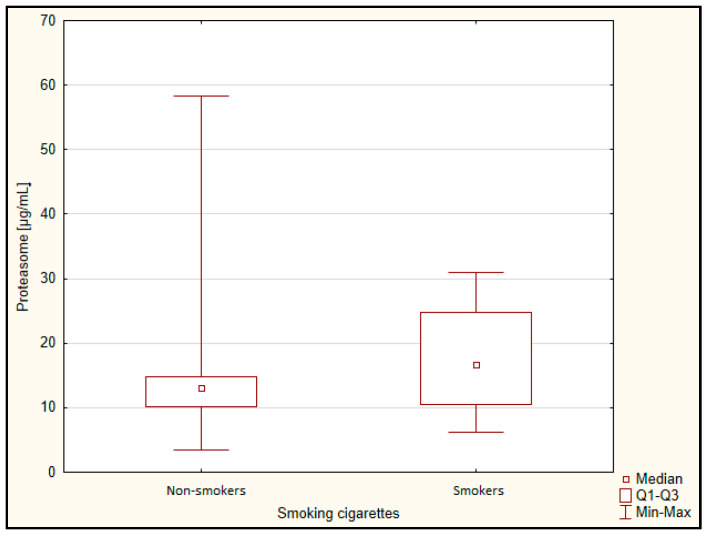
Concentration of proteasome depending on smoking cigarettes. Q1, lower quartile; Q3, upper quartile.

However, there was no correlation with the MMSE test result, as well as in the classification of patients into two groups (mild and moderate dementia) and five groups. There was also no correlation to the result of the clock drawing test, age, and BMI. There was no difference in the proteasome concentration between gender (Figure 3) and between smokers and non-smokers (Figure 4).

In the case of the proteasome concentration, no correlation was found with the concentration of mineral components (Se, Zn, Cu, and Cu/Zn) and TAS. There were also no differences between patients receiving various pharmacotherapy: rivastigmine and memantine (*p* = 0.910) and memantine and donepezil (*p* = 0.630).

There was a significant positive correlation between the concentration of UCHL1 and the concentration of the proteasome in the AD group, and a negative correlation in the control group (Table 6).

### 3.3. The Influence of Eating Habits on UCHL1 and Proteasome Concentration

The influence of eating habits on UCHL1 is presented in Table 7. A positive value of the β coefficient means that frequent consumption of a given product increases UCHL1; negative value that frequent consumption lowers this parameter. The adjusted value of R^2^ means that in this case 52% of the influence of the above factors was explained by the regression.

In the case of the proteasome, only two factors have a significant influence on the reduction or increase of its concentration, and the strength of the compound is small and amounts to 15% (Table 8).

## 4. Discussion

Contemporary literature data describe the ubiquitin-proteasome system as a potential therapeutic target in AD. This system is crucial for protein degradation in eukaryotes. Ubiquitin hydrolase (UCHL1) has been shown to increase the cellular levels of monoubiquitin and thus increase the protein turnover rate of the above system. Low levels of UCHL1 are associated with the accumulation of Aβ in AD [38].

UCHL1 is a deubiquitinating enzyme that is involved in the pathogenesis of neurodegenerative diseases, including AD. The main enzyme role is the elimination of misfolded proteins [39]. Our research allowed us to assess that AD patients are characterized by a higher median UCHL1 than healthy individuals (56.05 vs. 7.98 ng/mL, *p* < 0.000001). However, the decrease of this enzyme in brain tissue was observed in both ischemic injury and AD [40]. It is possible that the increased UCHL1 level in serum should compensate for the decrease in the enzyme concentration in brain tissue. A higher concentration of UCHL1 may also reflect the body’s metabolic response to acute inflammation; it is considered a biomarker for various forms of acute CNS damage [40].

Our research showed a tendency to have a negative link between the concentration of UCHL1 and Zn and between UCHL1 and TAS. Zinc deficiency has been shown to reduce UCHL1 expression in the rat hippocampus [41]. In contrast, studies on cultured hippocampal neurons showed that zinc modulated UCHL1 expression, suggesting that UCHL1 downregulation may be involved in memory dysfunction caused by a deficiency of this element [42].

Regression analysis showed that frequent consumption of honey, cooked vegetables, and milk may be negatively correlated with UCHL1 concentration, while consumption of fruit, other cold cuts (sirloin, ham), yellow and processed cheese, legumes, jams, and sausages may be positively correlated with this parameter. This group of products includes highly processed foods that are not recommended in the diet of AD patients. In 2017, a systematic review was published on the link between nutrition and AD. The authors found such a link in 50 of the 64 studies [43]. For example, a study of 5386 participants found a link between the consumption of trans fat, saturated fat, cholesterol, and total fat and the development of AD [44]. An excess of simple sugars and saturated fatty acids in the diet is a significant risk factor for AD. The so-called Western diet may result in, inter alia, the occurrence of obesity, dysbiosis of the intestinal microflora, and acceleration of low-grade systemic inflammation. These changes can lead to impairment of the blood-brain barrier and the development of neuroinflammation in addition to amyloid accumulation. The consequence may be dysfunction of synaptic transmission, neurodegeneration, and impairment of cognitive functions and memory [45].

In turn, the concentration of the proteasome may be positively affected by fruit consumption, and disadvantageously by the consumption of canned fish. Fish is rich in omega-3 fatty acids, including docosahexaenoic acid (DHA) and eicosapentaenoic acid (EPA). Shin et al. (2017) showed that DHA mediates intermolecular bonds of proteins through oxidation, and the resulting protein aggregates strongly reduce the activity of proteasomes—which has been proven both in vitro and in cultured cells. In cell models characterized by the overexpression of aggregation-prone proteins, such as for example tau, significantly increased levels of tau aggregates and total ubiquitin conjugates were found in the presence of DHA. In summary, DHA is described as a potent inducer of cellular protein aggregates that inhibit proteasome activity and retard systemic protein degradation in pathological conditions [46].

Other nutritional factors that are relevant to dementia and cognitive disorders include extra-virgin olive oil, nuts, berries, coffee, tea, cocoa, garlic, curcumin, omega-3 fatty acids, ginkgo biloba, resveratrol, phytoestrogens, and alcohol [47].

Donepezil and rivastigmine are cholinesterase inhibitors, while memantine is the N-methyl-d-aspartate receptor blocker [48]. In our study, we found no difference in UCHL1 levels in the rivastigmine and memantine groups compared to the memantine and donepezil groups. In the future, it is possible to conduct gene therapies in patients with impaired UCHL1 function.

The research by Öhrfelt et al. (2016) indicates that UCHL1 levels can be used as a cerebrospinal AD biomarker. The pilot study showed that the median UCHL1 in cerebrospinal fluid in the control group (*n* = 31) was 4.5 µg/L, while in the AD group (*n* = 10) it was significantly higher: 12 µg/L. This trend has been confirmed in the larger population. Finally, the control group was characterized by UCHL1 at the level of 7.2 µg/L, and the AD group at a significantly higher concentration—at the level of 11 µg/L [49]. Our research also showed a significant increase in UCHL1 in patients with AD.

Our results show that the SPRI technique with specific biosensors for the determination of UCHL1 and proteasome may be a very useful tool in the investigation of AD including effectiveness therapy, the recommended diet, etc.

## 5. Conclusions

Increased concentration of UCHL1 and the proteasome may be an important diagnostic marker of AD.

The increase in the above parameters can be caused by many factors, including eating habits. The result, inter alia, is different concentrations of individual components in the blood and tissues. Diet modifications by reducing the frequency of consumption of certain food categories, incl. highly processed foods may indirectly improve the clinical status of AD patients.

## Figures and Tables

**Table 1:** Characteristic of study groups.

Parameters	AD Group (*n* = 110)	Control Group (*n* = 60)
Gender		
F/M	80/30	46/14
	Age (years)	
Av. ± SD	78.0 ± 8.1	67.0 ± 7.9
Min.–Max	54–93	52–83
	BMI (kg/m^2^)	
Av. ± SD	26.5 ± 4.2	nd
Min.–Max	17.8–40.2	nd
	Habits	
Smoking cigarettes ^#^	13/97	nd
Alcohol drinking ^##^	5/105	nd
	MMSE (points)	
Av. ± SD	20.4 ± 4.3	nd
Min.–Max	11–26	nd

AD, Alzheimer’s disease; BMI, body mass index; F, female; M, male; MMSE, mini-mental state examination; *n*, number of subjects; nd, no data; SD, standard deviation, ^#^ 5–10 cigarettes/daily, ^##^ more than once a week.

**Table 2:** UCHL1 concentration [ng/mL] in the AD group compared to the control group.

	Control Group	AD Group	*p*
Av. ± SD	7.69 ± 1.76	55.75 ± 7.43	<0.000001 *
Min.–Max.	2.45–10.81	40.52–79.16	
Med.	7.98	56.05	
Q1	6.62	49.79	
Q3	9.33	61.04	

Av., mean; Med, median; Q1, lower quartile; Q3, upper quartile; SD, standard deviation. * was supposed to mean statistical significance.

**Table 3:** Significant correlations between the concentration of UCHL1 and selected parameters.

Parameter 1	Parameter 2	r	*p*
UCHL1	Zn	−0.338	<0.000004 *
	TAS	−0.217	<0.01 *
	Cu/Zn	0.262	<0.002 *

Av., mean; Med, median; Q1, lower quartile; Q3, upper quartile; SD, standard deviation; TAS, Total Antioxidant Status. * was supposed to mean statistical significance.

**Table 4:** UCHL1 concentration [ng/mL] depending on pharmacotherapy.

	Rivastigmine + Memantine	Memantine + Donepezil
Av. ± SD	60.44 ± 4.06	56.73 ± 5.56
Min.–Max.	55.31–65.34	43.17–63.86
Med.	59.16	58.46
Q1	58.65	55.34
Q3	63.71	61.04
	**No pharmacotherapy**	**No pharmacotherapy**
Av. ± SD	55.51 ± 7.99	55.53 ± 7.80
Min.–Max.	40.52–79.15	40.52–79.15
Med.	55.88	55.88
Q1	49.26	49.17
Q3	60.85	60.95
*p*	0.16	0.41

Av., mean; Med, median; Q1, lower quartile; Q3, upper quartile; SD, standard deviation.

**Table 5:** Proteasome concentration [µg/mL] in the test group compared to the control group.

	Control Group	AD Group	*p*
Av. ± SD	5.20 ± 1.82	13.94 ± 9.88	<0.0001 *
Min.–Max.	1.90–7.86	3.46–60.47	
Med.	5.72	13.02	
Q1	3.13	9.84	
Q3	6.34	15.04	

Av., mean; Med, median; Q1, lower quartile; Q3, upper quartile; SD, standard deviation. * was supposed to mean statistical significance.

**Table 6:** Significant correlations between the concentration of UCHL1 and proteasome.

Parameter 2	r	*p*
Control group	−0.372	*p* < 0.02 *
AD group	0.266	*p* < 0.007 *

AD: Alzheimer’s disease, r: correlation coefficient. * was supposed to mean statistical significance.

**Table 7:** Stepwise multiple linear regression analysis of the influence of the frequency of consuming food products on the concentration of UCHL1.

Independent Variables	β Coefficient (SE)	Significance Level	Adjusted R^2^
Honey	**−0.603 (0.138)**	**0.0001**	
Cooked vegetables	**−0.306 (0.103)**	**0.0047**	**0.52**
Milk	**−0.252 (0.114)**	**0.0318**	
Fruit	**0.405 (0.111)**	**0.0007**	
Other cold cuts (ham, sirloin)	**0.311 (0.102)**	**0.0040**	
Yellow and processed cheeses	**0.289 (0.116)**	**0.0169**	
Legumes	**0.266 (0.111)**	**0.0215**	
Jams	**0.263 (0.117)**	**0.0302**	
Sausages	**0.260 (0.115)**	**0.0289**	
Coffee	−0.169 (0.111)	0.1360	
Oils	−0.166 (0.102)	0.1128	
Tea	0.109 (0.103)	0.2971	

Statistically significant products (*p* < 0.05) are marked in bold.

**Table 8:** Stepwise multiple linear regression analysis of the influence of the frequency of consuming food products on the concentration of proteasome.

Independent Variables	β Coefficient (SE)	Significance Level	Adjusted R^2^
Canned fish	**−0.283 (0.124)**	**0.0266**	**0.15**
Fruit	**0.284 (0.134)**	**0.0383**	
White cheeses	0.171 (0.126)	0.1805	
Sausages	0.169 (0.131)	0.2025	
Fish	−0.137 (0.131)	0.2994	
Honey	−0.186 (0.134)	0.1702	

Statistically significant products (*p* < 0.05) are marked in bold.

## Data Availability

The data presented in this study are available on request from the corresponding author. The data are not publicly available due to privacy of ethical.

## References

[1] Sobów T. (2010). Praktyczna Psychogeriatria—Rozpoznawanie i Postępowanie w Zaburzeniach Psychicznych u Chorych w Wieku Podeszłym [Practical Psychogeriatry—Diagnosis and Management of MENTAL disorders in Elderly Patients].

[2] Masliah E., Ellisman M., Carragher B., Mallory M., Young S., Hansen L., DeTeresa R., Terry R.D. (1992). Three-dimensional analysis of the relationship between synaptic pathology and neuropil threads in Alzheimer disease. J. Neuropathol. Exp. Neurol..

[3] Rowland N.C., Sammartino F., Tomaszczyk J.C., Lozano A.M. (2016). Deep Brain Stimulation of the Fornix: Engaging Therapeutic Circuits and Networks in Alzheimer Disease. Neurosurgery.

[4] Urbaniak A. (2012). Receptor p75NTR—Rola w procesach wzrostu i śmierci komórki [P75NTR receptor—Role in the processes of cell growth and death]. Postepy Hig. Med. Dosw..

[5] Tarawneh R., Holtzman D.M. (2012). The clinical problem of symptomatic Alzheimer disease and mild cognitive impairment. Cold Spring Harb. Perspect. Med..

[6] Do H.A., Baek K.H. (2021). Cellular functions regulated by deubiquitinating enzymes in neurodegenerative diseases. Ageing Res. Rev..

[7] Liu B., Ruan J., Chen M., Li Z., Manjengwa G., Schlüter D., Song W., Wang X. (2021). Deubiquitinating enzymes (DUBs): Decipher underlying basis of neurodegenerative diseases. Mol. Psychiatry.

[8] Hommen F., Bilican S., Vilchez D. (2021). Protein clearance strategies for disease intervention. J. Neural Transm..

[9] Hong L., Huang H.C., Jiang Z.F. (2014). Relationship between amyloid-beta and the ubiquitin-proteasome system in Alzheimer’s disease. Neurol. Res..

[10] Day I.N., Thompson R.J. (2010). UCHL1 (PGP 9.5): Neuronal biomarker and ubiquitin system protein. Prog. Neurobiol..

[11] Doran J.F., Jackson P., Kynoch P.A., Thompson R.J. (1983). Isolation of PGP 9.5, a new human neurone-specific protein detected by high-resolution two-dimensional electrophoresis. J. Neurochem..

[12] Matuszczak E., Tylicka M., Komarowska M.D., Debek W., Hermanowicz A. (2020). Ubiquitin carboxy-terminal hydrolase L1—Physiology and pathology. Cell Biochem. Funct..

[13] Zhang M., Cai F., Zhang S., Song W. (2014). Overexpression of ubiquitin carboxyl-terminal hydrolase L1 (UCHL1) delays Alzheimer’s progression in vivo. Sci. Rep..

[14] Jara J.H., Genç B., Cox G.A., Bohn M.C., Roos R.P., Macklis J.D., Ulupınar E., Özdinler P.H. (2015). Corticospinal Motor Neurons Are Susceptible to Increased ER Stress and Display Profound Degeneration in the Absence of UCHL1 Function. Cereb Cortex.

[15] Lederer C.W., Torrisi A., Pantelidou M., Santama N., Cavallaro S. (2007). Pathways and genes differentially expressed in the motor cortex of patients with sporadic amyotrophic lateral sclerosis. BMC Genom..

[16] Chu K.Y., Li H., Wada K., Johnson J.D. (2012). Ubiquitin C-terminal hydrolase L1 is required for pancreatic beta cell survival and function in lipotoxic conditions. Diabetologia.

[17] Matuszczak E., Tylicka M., Dębek W., Sankiewicz A., Gorodkiewicz E., Hermanowicz A. (2017). Overexpression of ubiquitin carboxyl-terminal hydrolase L1 (UCHL1) in serum of children after thermal injury. Adv. Med. Sci..

[18] Kornitzer D., Ciechanover A. (2000). Modes of regulation of ubiquitin-mediated protein degradation. J. Cell Physiol..

[19] Keller J.N., Hanni K.B., Markesbery W.R. (2000). Impaired proteasome function in Alzheimer’s disease. J. Neurochem..

[20] López Salon M., Morelli L., Castaño E.M., Soto E.F., Pasquini J.M. (2000). Defective ubiquitination of cerebral proteins in Alzheimer’s disease. J. Neurosci. Res..

[21] Ito W.D., Lund N., Zhang Z., Buck F., Lellek H., Horst A., Machens H.G., Schunkert H., Schaper W., Meinertz T. (2015). Activation of Cell Surface Bound 20S Proteasome Inhibits Vascular Cell Growth and Arteriogenesis. Biomed. Res. Int..

[22] Majetschak M., Perez M., Sorell L.T., Lam J., Maldonado M.E., Hoffman R.W. (2008). Circulating 20S proteasome levels in patients with mixed connective tissue disease and systemic lupus erythematosus. Clin. Vaccine Immunol..

[23] Henry L., Lavabre-Bertrand T., Douche T., Uttenweiler-Joseph S., Fabbro-Peray P., Monsarrat B., Martinez J., Meunier L., Stoebner P.E. (2010). Diagnostic value and prognostic significance of plasmatic proteasome level in patients with melanoma. Exp. Dermatol..

[24] Jakob C., Egerer K., Liebisch P., Türkmen S., Zavrski I., Kuckelkorn U., Heider U., Kaiser M., Fleissner C., Sterz J. (2007). Circulating proteasome levels are an independent prognostic factor for survival in multiple myeloma. Blood.

[25] Tylicka M., Matuszczak E., Dębek W., Hermanowicz A., Ostrowska H. (2014). Circulating proteasome activity following mild head injury in children. Childs Nerv. Syst..

[26] Ding Q., Keller J.N. (2003). Does proteasome inhibition play a role in mediating neuropathology and neuron death in Alzheimer’s disease?. J. Alzheimers Dis..

[27] Keller J.N., Markesbery W.R. (2000). Proteasome inhibition results in increased poly-ADP-ribosylation: Implications for neuron death. J. Neurosci. Res..

[28] Kopito R.R. (2000). Aggresomes, inclusion bodies and protein aggregation. Trends Cell Biol..

[29] Lam Y.A., Pickart C.M., Alban A., Landon M., Jamieson C., Ramage R., Mayer R.J., Layfield R. (2000). Inhibition of the ubiquitin-proteasome system in Alzheimer’s disease. Proc. Natl. Acad. Sci. USA.

[30] van Leeuwen F.W., Burbach J.P., Hol E.M. (1998). Mutations in RNA: A first example of molecular misreading in Alzheimer’s disease. Trends Neurosci..

[31] McKhann G.M., Knopman D.S., Chertkow H., Hyman B.T., Jack C.R., Kawas C.H., Klunk W.E., Koroshetz W.J., Manly J.J., Mayeux R. (2011). The diagnosis of dementia due to Alzheimer’s disease: Recommendations from the National Institute on Aging-Alzheimer’s Association workgroups on diagnostic guidelines for Alzheimer’s disease. Alzheimers Dement..

[32] Socha K., Klimiuk K., Naliwajko S.K., Soroczyńska J., Puścion-Jakubik A., Markiewicz-Żukowska R., Kochanowicz J. (2021). Dietary Habits, Selenium, Copper, Zinc and Total Antioxidant Status in Serum in Relation to Cognitive Functions of Patients with Alzheimer’s Disease. Nutrients.

[33] Sankiewicz A., Laudanski P., Romanowicz L., Hermanowicz A., Roszkowska-Jakimiec W., Debek W., Gorodkiewicz E. (2015). Development of surface plasmon resonance imaging biosensors for detection of ubiquitin carboxyl-terminal hydrolase L1. Anal. Biochem..

[34] Sankiewicz A., Romanowicz L., Pyc M., Hermanowicz A., Gorodkiewicz E. (2018). SPR imaging biosensor for the quantitation of fibronectin concentration in blood samples. J. Pharm. Biomed. Anal..

[35] Sankiewicz A., Romanowicz L., Laudanski P., Zelazowska-Rutkowska B., Puzan B., Cylwik B., Gorodkiewicz E. (2016). SPR imaging biosensor for determination of laminin-5 as a potential cancer marker in biological material. Anal. Bioanal. Chem..

[36] Gorodkiewicz E., Ostrowska H., Sankiewicz A. (2011). SPR imaging biosensor for the 20S proteasome: Sensor development and application to measurement of proteasomes in human blood plasma. Mikrochim. Acta.

[37] Falkowski P., Mrozek P., Lukaszewski Z., Oldak L., Gorodkiewicz E. (2021). An Immunosensor for the Determination of Cathepsin S in Blood Plasma by Array SPRi-A Comparison of Analytical Properties of Silver-Gold and Pure Gold Chips. Biosensors.

[38] Gong B., Radulovic M., Figueiredo-Pereira M.E., Cardozo C. (2016). The Ubiquitin-Proteasome System: Potential Therapeutic Targets for Alzheimer’s Disease and Spinal Cord Injury. Front. Mol. Neurosci..

[39] Guglielmotto M., Monteleone D., Vasciaveo V., Repetto I.E., Manassero G., Tabaton M., Tamagno E. (2017). The Decrease of Uch-L1 Activity Is a Common Mechanism Responsible for Aβ 42 Accumulation in Alzheimer’s and Vascular Disease. Front. Aging Neurosci..

[40] Wang K.K., Yang Z., Sarkis G., Torres I., Raghavan V. (2017). Ubiquitin C-terminal hydrolase-L1 (UCH-L1) as a therapeutic and diagnostic target in neurodegeneration, neurotrauma and neuro-injuries. Expert Opin. Ther. Targets.

[41] Gong B., Cao Z., Zheng P., Vitolo O.V., Liu S., Staniszewski A., Moolman D., Zhang H., Shelanski M., Arancio O. (2006). Ubiquitin hydrolase Uch-L1 rescues beta-amyloid-induced decreases in synaptic function and contextual memory. Cell.

[42] Liu J., Jiang Y.G., Huang C.Y., Fang H.Y., Fang H.T., Pang W. (2008). Depletion of intracellular zinc down-regulates expression of Uch-L1 mRNA and protein, and CREB mRNA in cultured hippocampal neurons. Nutr. Neurosci..

[43] Yusufov M., Weyandt L.L., Piryatinsky I. (2017). Alzheimer’s disease and diet: A systematic review. Int. J. Neurosci..

[44] Kalmijn S., Launer L.J., Ott A., Witteman J.C., Hofman A., Breteler M.M. (1997). Dietary fat intake and the risk of incident dementia in the Rotterdam Study. Ann. Neurol..

[45] Więckowska-Gacek A., Mietelska-Porowska A., Wydrych M., Wojda U. (2021). Western diet as a trigger of Alzheimer’s disease: From metabolic syndrome and systemic inflammation to neuroinflammation and neurodegeneration. Ageing Res. Rev..

[46] Shin S.K., Kim J.H., Lee J.H., Son Y.H., Lee M.W., Kim H.J., Noh S.A., Kim K.P., Kim I.G., Lee M.J. (2017). Docosahexaenoic acid-mediated protein aggregates may reduce proteasome activity and delay myotube degradation during muscle atrophy in vitro. Exp. Mol. Med..

[47] Dominguez L.J., Veronese N., Vernuccio L., Catanese G., Inzerillo F., Salemi G., Barbagallo M. (2021). Nutrition, Physical Activity, and Other Lifestyle Factors in the Prevention of Cognitive Decline and Dementia. Nutrients.

[48] Jagaran K., Singh M. (2021). Nanomedicine for Neurodegenerative Disorders: Focus on Alzheimer’s and Parkinson’s Diseases. Int. J. Mol. Sci..

[49] Öhrfelt A., Johansson P., Wallin A., Andreasson U., Zetterberg H., Blennow K., Svensson J. (2016). Increased Cerebrospinal Fluid Levels of Ubiquitin Carboxyl-Terminal Hydrolase L1 in Patients with Alzheimer’s Disease. Dement. Geriatr. Cogn. Dis. Extra.

